# Medical News

**Published:** 1885-10

**Authors:** 


### Medical News.

There is every prospect that small-pox will prevail epidemically in some parts of the United States. The number of cases in Montreal is increasing, there having been 218 deaths in one week, ending September 19th. The disease has broken out in New York City, on Grand street, a thickly populated tenement district, and in Brooklyn. In foreign countries, small-pox is prevalent in London, Paris, Bordeaux, Lisbon and Venice. During the cold weather in our climate, this disease is more easily propagated than in summer, consequently our local Board of Health must be unusually active if they would avoid an epidemic of variola this winter.

---

The cholera in southern Europe appears to be spreading, but the disease is not so virulent as at the beginning of the epidemic. The disease has reached Gibraltar, on the west, and has again broken out in Italy, at Parma and Palermo. In the latter place, the people are panic-stricken. Thirty thousand people have fled from their homes, while those who remain wage a continual war with the sanitary authorities to prevent the disinfection of plague-stricken districts.

The third annual meeting of the American Rhinological Association will be held at Lexington, Ky., October 6, 1885. Papers and discussion will be devoted exclusively to the diseases of the nasal passages and their sequences. Officers for 1885: President, P. W. Logan, M.D., Knoxville, Tenn.; First Vice-President, A. De Vilbiss, M.D., Toledo, Ohio; Second Vice-President, J. A. Stucky, M.D., Lexington, Ky.; Recording Secretary, C. A. Sims, M.D., St. Joseph, Mo.; Librarian, N. R. Gordon, M.D., Springfield, Ill. Council: J. G. Carpenter, M.D., Stanford, Ky.; H. Jerard, M.D., East Lynne, Mo.; H. Christopher, M.D., St. Joseph, Mo.; E. F. Henderson, M.D., Los Angeles, Cal.

---

Yellow fever, which has been prevalent in Havana during the summer, has reached Vera Cruz, Mexico. It is stated that there is an unusual amount of emigration northward from this place, through El Paso, Texas. The government authorities have ordered the inspection of all trains and baggage, to prevent the introduction of the disease into the country.

---

The death of Wm. A. Grey, M.B., F.R.S., Prof. of Hygiene in King's College, London, and author of the well-known text-book, "The Principles of Forensic Medicine," is reported.

---

At the meeting of the International Medical Congress, in Copenhagen, an estimate of the number of physicians in the entire world was made. The number was 189,650.

---

In the Lying-in-Hospital of Vienna, 9,000 women are confined annually. The rate of mortality is one-half per cent. for the mothers and 15 per cent. for the children.

